# Pre-movement muscle co-contraction associated with motor performance deterioration under high reward conditions

**DOI:** 10.1038/s41598-024-67630-5

**Published:** 2024-07-19

**Authors:** Naoki Senta, Junichi Ushiba, Mitsuaki Takemi

**Affiliations:** 1 Graduate School of Science and Technology, Keio University, 3-14-1 Hiyoshi, Kohoku-ku, Yokohama, Kanagawa 223-8522 Japan; 2 Faculty of Science and Technology, Keio University, Yokohama, Japan

**Keywords:** Motor control, Biomarkers

## Abstract

Reward usually enhances task performance, but exceptionally large rewards can impede performance due to psychological pressure. In this study, we investigated motor activity changes in high-reward situations and identified indicators for performance decline. Fourteen healthy adults practiced a velocity-dependent right-hand motor task for three days, followed by a test day with varying monetary reward for each trial. Participants were divided into low performers (LPs) and high performers (HPs) according to whether success rate decreased or increased, respectively, on the highest reward trials compared to lower reward trials. Both LPs and HPs demonstrated increased hand velocity during higher reward trials, but only LPs exhibited a significant increase in velocity variance. There was also a negative correlation between the pre-movement co-contraction index (CCI) of the biceps and triceps muscles and success rate on the highest reward trials. This correlation was confirmed in a second experiment with 12 newly recruited participants, suggesting that pre-movement CCI is a marker for performance decline caused by high reward. These findings suggest that interventions to reduce pre-movement CCI such as biofeedback training could be useful for preventing the paradoxical decline in motor performance associated with high rewards.

## Introduction

Rewards may improve task performance by enhancing motivation, directing attention, and promoting engagement. When rewards are appropriately aligned with the task and provide a sense of accomplishment and recognition, they can inspire individuals to strive for excellence^1–4^. Rewards have been shown to enhance motor performance by reducing response latency and increasing the speed, accuracy, and stability of movements such as the hand reaching to a target frequently required for sport and recreation^4^. Additionally, negative rewards have been shown to accelerate learning speed, while positive rewards improve the retention of motor memory^5^. Despite these benefits, rewards can also impair motor performance under certain conditions. For example, large rewards may create undue pressure and anxiety, resulting in diminished performance (“choking under pressure”). Moreover, large rewards can lead to distractions, excessive monitoring, and over-arousal^6–8^. These performance impediments may emerge in critical singular situations such as golf-putting^9–11^, football penalty kicks^12^, and math problem-solving^13–15^.

A decline in motor performance due to psychological pressure has been widely documented in various laboratory motor tasks. For instance, Baumeister et al.^16^ reported that scores on a game task requiring complex dexterity decreased under the psychological pressure incurred by implicit competition, a monetary incentive and audience. Similarly, Mobbs et al.^17^ found that high monetary rewards increased the probability of tracking failure in an object chasing task requiring swift reactions. Performance disruptions due to high rewards have also been observed in upper limb motor tasks commonly employed in motor control research^18–20^. The extent of reward-induced performance reduction has been linked to neural activity in the midbrain and striatum, indicating that brain regions encoding motivation associated with rewards play a crucial role in poor performance under pressure^17,18^. In addition, functional connectivity between the dorsolateral prefrontal cortex and motor cortex before the movement onset was negatively related to performance decrement, suggesting a compensatory mechanism in which top–down control of the prefrontal cortex over motor functions alleviates the adverse effects of pressure^20^.

While the neural responses to reward have been studied for many decades, the reward-dependent changes in muscle activity directly generating motor outputs remain largely unexplored, especially in humans. In nonhuman primates, it was found that arm-reaching movements are more likely to undershoot the target when the reward amount increases^21^. Identify changes in muscle activity that predict task failure due to excessive reward may provide guidance for the development of simple biosignal measurement systems or even training procedures to reduce performance decrements under psychological pressure during real-world settings, such as competitive sports.

In the current study, we investigated how increases in monetary reward alter motor task performance and associated muscle activity. To this end, we designed a video-based laboratory motor task that models sports activities such as golf, wherein the velocity of right-hand movement at the time of impact with an object (ball) determines target placement (outcome). A group of healthy adults practiced the task for three days and performance was tested on the fourth day with different reward amounts (1, 10, 100, or 1000 yen) per trial while simultaneously recording the surface electromyogram (EMG) from the right upper limb. Participants were then divided into high- and low-performance groups depending on whether success rate increased or decreased, respectively, on high-reward trials (1000 yen) compared to lower reward trials (100 yen). We compared changes in object hitting speed, speed variability, and muscle co-contraction indices (CCIs) calculated from the EMG signals between reward conditions and between groups. We then evaluated the correlations between performance scores and CCI in the highest reward condition. Finally, to confirm replicability of these findings and address potential confounding factors, a similar experiment was conducted with 12 new participants.

## Results

### Velocity control task performance throughout experiment

**Figure 1:**
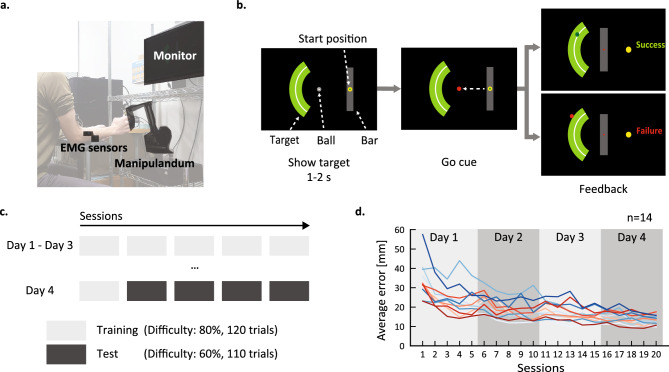
The velocity control task used to assess the effects of reward size on motor performance and muscle activity. (**a**) Experimental setup. Participants sat 0.6 m away from the screen and operated the manipulandum to control a bar on the monitor. (**b**) Participants moved the manipulandum handle to control the gray bar on the screen. They were instructed to hit the ball as close as possible to the target (green arch) by moving the bar, and were then informed that the impact speed determines the distance traveled by the ball. For individual trials, participants held the bar in the starting position while the target was presented. After 1–2 s, the ball color changed to red, and the participant moved the bar and hit the ball immediately. When the ball stopped, performance feedback was displayed. (**c**) Experimental procedure. The experiment was conducted over four days. The first three days consisted of 5 training sessions per day with 120 trials per session. Task difficulty was adjusted during each session to achieve 80% success by manipulating the target size according to performance of the previous 10 trials and the previous session. On the fourth day, participants performed a single training session, followed by four test sessions consisting of 110 trials per session. Test task difficulty was adjusted individually according to performance on sessions 11 through 16 to achieve an average 60% success rate but target size was constant throughout the test sessions. (**d**) Time course of mean endpoint error averaged over trials within a session. Endpoint error is defined as the distance between the ball endpoint and the centerline of the target. Each line represents the performance of one individual. Participants were then categorized as high performers (red lines) or low performers (blue lines) as described in the “Methods” section.

In the first experiment, we investigated how reward amount influences motor task performance and associated muscle activity. Fourteen healthy young adults performed a velocity control task that involved moving the right hand at a precise speed, similar to real-world sports such as golf. The task required participants to manipulate a bar displayed on a screen using a robot manipulandum (Fig. 1a, with the goal of hitting a virtual ball in the center of the screen and placing it in a target area to the left (Fig. 1b). The experiment was conducted over four consecutive days (Fig. 1c). During the first three days, participants performed five training sessions per day. As a result of training, participants exhibited a convergence of performance, with the endpoint of the ball (placement) moving progressively closer to the center of the target (Fig. 1d). There was also a gradual improvement in consistency as measured by error distance variation (Supplementary Fig. 1).

### Performance modification by monetary reward and classification of high and low performers

**Figure 2:**
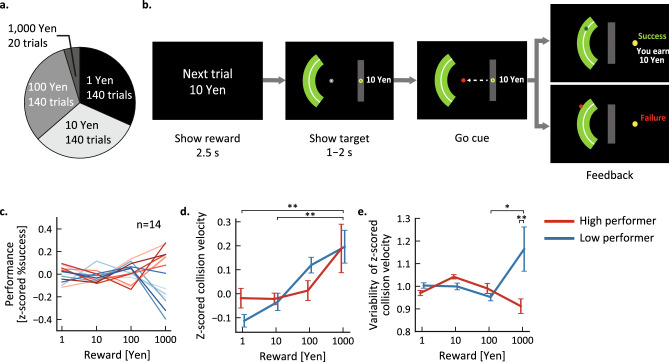
Low performers exhibit greater variability in normalized collision velocity than high performers on test trials with highest reward. (**a**) Relative frequency of each reward in test trials. Trials with the largest reward (1000 yen) occurred less often than trials with lower rewards. (**b**) Task procedure. The reward amount was presented on the right side of the monitor during the trial. (**c**) Task performance. The success rate was z-scored for each participant. Each line represents the performance of a single participant, with blue lines representing low performers (participants with reduced success rate on 1000 yen trials compared to 100 yen trials) and red lines representing high performers (who performed better on 1000 yen trials). (**d**) Averaged hand velocity and (**e**) variability of hand velocity (standard deviation) at the instance of ball collision. Lines represent the mean ± SEM. *p < 0.05; **p < 0.01.

On the fourth day of the experiment, a single training session was followed by four testing sessions (Fig. 1c). Participants were provided with one of four monetary rewards (1, 10, 100, or 1000 yen) for successfully completing each trial (Fig. 2a). In this experiment, the 1000 yen condition (highest reward) was comparatively less frequent than the other reward conditions. Prior to the start of each trial, the participants were informed about the specific monetary reward that could be earned (Fig. 2b).

Motor performance variability was substantially greater across participants in highest reward trials (1000 yen) compared to lower reward trials (Fig. 2c, Table 1). To assess the effect of reward amount on task performance, it was first necessary to compute performance Z-scores for each individual according to overall average performance^18^. Even with this normalization for individual ability, there was no consistent change in performance across all participants as reward increased (*F*(1.62, 21.1) = 0.15, *p* = 0.82). However, reward can either improve performance by increasing motivation or decrease performance by creating psychological pressure^22^. Thus, to compare cases where rewards had a positive or negative effect, we divided participants into high performer (HP) and low performer (LP) subgroups according to whether performance on 1000 yen trials was better or worse, respectively, than on 100 yen trials. Based on this criterion, seven participants were classified as HPs and the remaining seven participants as LPs.

**Table 1:** Changes in motor performance and kinematics of the first experiment.

Reward [yen]	Subgroup	Performance [z-scored %success]	Z-scored collision velocity	Variability of z-scored collision velocity
1	HP	6.3 × 10^−3^ (0.068)	− 0.017 (0.12)	0.97 (0.033)
	LP		− 0.11 (0.069)	1.0 (0.032)
10	HP	− 0.017 (0.052)	− 8.6 × 10^−3^ (0.059)	1.0 (0.027)
	LP		− 0.034 (0.096)	1.0 (0.039)
100	HP	0.013 (0.083)	2.3 × 10^−3^ (0.12)	0.99 (0.063)
	LP		0.12 (0.087)	0.95 (0.043)
1000	HP	− 0.012 (0.23)	0.16 (0.28)	0.90 (0.088)
	LP		0.20 (0.18)	1.2 (0.26)

Performance and kinematic change values are presented as the mean and standard deviation.

Success or failure on each trial was determined by hand velocity (and by extension bar velocity on the monitor) at the time of impact with the virtual ball, and success rate was reduced when the average velocity deviated from the ideal velocity, so overall accuracy was reduced when the variability in velocity was higher. Therefore, we investigated the kinematic changes underlying reward-induced performance changes (Table 1). The results of analysis of variance (ANOVA) reward condition (amount) as the within-subject factor and group (LP or HP) as the between-subject factor revealed that mean hand velocity when hitting the ball increased with reward, while the variability of hand velocity (defined as the standard deviation) increased only in the 1000 yen condition among the LP participants. Specifically, the ANOVA revealed a significant main effect of reward on mean hand velocity (*F*(3, 36) = 8.26, *p* < 0.001), and post-hoc tests (using Bonferroni correction for multiple comparisons) revealed significantly greater average hand velocity in the 1000 yen trials compared to 1 yen and 10 yen trials (Fig. 2d, 1- vs. 1000-yen: *p* < 0.001; 10- vs. 1000-yen: *p* = 0.002). Conversely, there was no significant main effect of subgroup (*F*(1, 12) = 0.004, *p* = 0.95) or interaction between reward and subgroup (*F*(3, 36) = 1.063, *p* = 0.38). For variability of hand velocity, however, there was a significant main effect of subgroup (*F*(1, 12) = 6.22, *p* = 0.028) and a significant reward × subgroup interaction (*F*(1.25, 14.97) = 5.62, *p* = 0.026). Post-hoc tests further revealed that velocity variability among LP participants was significantly greater in 1000 yen trials than 100 yen trials (*p* = 0.025) and significantly greater than during 1000 yen trials among HP participants (*p* < 0.001) (Fig. 2e). Alternatively, there was no significant main effect of reward amount (*F*(1.25, 14.97) = 1.09, *p* = 0.33).

### Muscular co-contraction during pre-movement and movement phases is differentially influenced by reward amount among high and low performers

**Figure 3:**
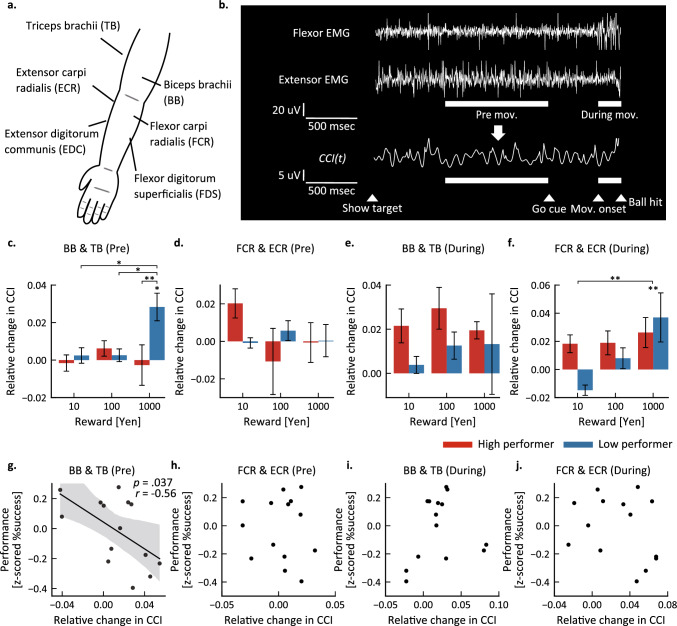
High reward increases pre-movement muscle co-contraction among low performers. (**a**) Sites of electromyogram (EMG) recording. (**b**) Calculation of the muscle co-contraction index (CCI). *Top*: EMG waveforms from the elbow flexor (biceps brachii, BB) and extensor (triceps brachii, TB) muscles. *Bottom*: CCI changes from target presentation to ball hit derived from the flexor and extensor EMGs. The two white bars below the waveforms represent the two analysis periods: the 1000-ms pre-movement (Pre) period before the Go cue, and from movement onset to the ball hit (During). See “Methods” section for the CCI formula. (**c**–**f**) Changes in CCI for BB and TB muscles and for flexor carpi radialis and extensor carpi radialis (FCR & ECR) muscles within the pre-movement period (Pre) and movement period (During). Results for both HP and LP groups are shown as mean ± SEM. *p < 0.05; **p < 0.01. (**g**–**j**) Scatter plots of CCI versus task success rate for 1000 yen trials. The regression line and 95% confidence interval (shaded area) are shown for the significant correlation of BB-TB CCI with success rate (**g**). Other correlations were not statistically significant.

We measured EMG signals from six upper limb muscles (Fig. 3a) and investigated changes in co-contraction of three different antagonistic muscle pairs during pre-movement and movement phases of velocity control task trials (Fig. 3b, Table 2). During pre-movement, the CCI of the biceps brachii (BB) and triceps brachii (TB) pair (BB-TB) was significantly greater during highest reward (1000 yen) trials in the LP group but not the HP group (Fig. 3c). Notably, two-way ANOVA revealed a significant interaction between reward and subgroup (LP vs. HP) for BB-TB CCI during the pre-movement period (*F*(1.54, 18.4) = 4.61, *p* = 0.032), and subsequent post-hoc tests indicated that pre-movement BB-TB CCI was significantly higher in 1000 yen trials compared to 1, 10, and 100 yen trials among LP participants (1- vs. 1000-yen: *p* = 0.013; 10- vs. 1000-yen: *p* = 0.032; 100- vs. 1000-yen: *p* = 0.034). There was also a significant difference in pre-movement BB-TB CCI between HP and LP groups during the 1000 yen trials (*p* = 0.005). Conversely, there were no significant main effects or interaction effects of group and reward on the CCI of flexor carpi radialis (FCR) and extensor carpi radialis (ECR) muscles or flexor digitorum superficialis (FDS) and extensor digitorum communis (EDC) muscles during pre-movement (Fig. 3d and Supplementary Fig. 2a).

**Table 2:** Relative changes in CCI in the first experiment.

Reward [yen]	Subgroup	BB & TB	FCR & ECR	FDS & EDC
Pre-movement				
10	HP	− 1.5 × 10^–3^ (0.012)	0.020 (0.021)	6.5 × 10^–3^ (0.012)
	LP	2.4 × 10^–3^ (0.011)	− 8.4 × 10^–4^ (7.3 × 10^–3^)	4.3 × 10^–3^ (6.7 × 10^–3^)
100	HP	6.2 × 10^–3^ (0.011)	-0.011 (0.047)	− 5.9 × 10^–3^ (0.046)
	LP	2.6 × 10^–3^ (8.9 × 10^–3^)	5.7 × 10^–3^ (0.014)	8.3 × 10^–3^ (0.018)
1000	HP	-2.7 × 10^–3^ (0.029)	-6.4 × 10^–4^ (0.028)	-0.016 (0.049)
	LP	0.028 (0.019)	3.8 × 10^–4^ (0.023)	8.4 × 10^–3^ (0.022)
During movement				
10	HP	0.022 (0.020)	0.018 (0.017)	− 2.7 × 10^–3^ (0.014)
	LP	0.004 (0.010)	− 0.015 (0.010)	− 4.6 × 10^–4^ (0.019)
100	HP	0.029 (0.025)	0.019 (0.022)	0.018 (0.025)
	LP	0.013 (0.016)	8.0 × 10^–3^ (0.020)	0.017 (0.022)
1000	HP	0.019 (0.010)	0.026 (0.028)	0.025 (0.058)
	LP	0.013 (0.060)	0.037 (0.046)	0.025 (0.040)

CCI is presented as the mean and standard deviation.

There were no significant main effects or a group × reward interaction effect on BB-TB CCI and FDS-EDC CCI during movement (Fig. 3e and Supplementary Fig. 2b). In contrast, reward amounts significantly increased the FCR-ECR CCI during movement in both groups (Fig. 3f). Accordingly, there was a significant main effect of reward on FCR-ECR CCI during movement (*F*(1.65, 19.7) = 6.16, *p* = 0.011), but no group × reward interaction (*F*(1.65, 19.7) = 2.54, *p* = 0.11). Post-hoc tests demonstrated an increase in the FCR-ECR CCI for the 1000 yen condition compared to the 1 and 10 yen conditions (1- vs. 1000-yen: *p* = 0.003; 10- vs. 1000-yen: *p* = 0.006). Additional statistical test results are presented in Supplementary Table 1.

We then examined whether the CCI change for each participant was correlated with individual performance. Consistent with the difference between HP and LP groups, BB-TB CCI during pre-movement in 1000 yen trials was negatively correlated with performance (Fig. 3g; *r* =  − 0.56, *p* = 0.037), while no significant correlations were found for FCR-ECR CCI during pre-movement (Fig. 3h; *r*(12) =  − 0.09, *p* = 0.76), BB-TB CCI during movement (Fig. 3i; *r*(12) = 0.18, *p* = 0.53), and FCR-ECR CCI during movement (Fig. 3j; *r*(12) =  − 0.28, *p* = 0.33). Furthermore, no significant correlation was observed between pre-movement BB-TB CCI and performance in conditions other than the highest reward condition. Specifically, correlation analysis revealed no significant correlation in the 10- and 100-yen conditions (10 yen: *r*(12) = 0.38, *p* = 0.26; 100 yen: *r*(12) =  − 0.22, *p* = 0.46; Supplementary Fig. 3a,b).

### ECG recordings indicate a link between reward-induced changes in motor performance and sympathetic dominance

We then examined if these changes in motor task performance and muscle activity (particularly pre-movement co-contraction) during high-reward trials are related to the stress response by measuring the electrocardiogram (ECG). From these recordings, we calculated the heart rate variability (HRV) indices normalized high-frequency power (nHF), which is widely believed to reflect parasympathetic dominance in cardiovascular autonomic inputs, and both normalized low-frequency power (nLF) and the ratio of low-frequency to high-frequency power (LF/HF), which are believed to be indicators of sympathetic dominance^23,24^.

**Figure 4:**
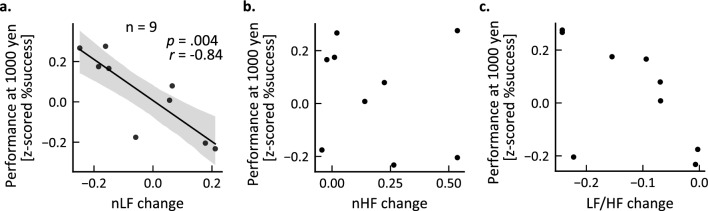
Potential involvement of sympathetic nervous system overactivation in poor performance on high-reward trials. (**a**–**c**) Correlations between relative changes in heart rate variability (HRV) indices derived from ECG recordings and task success rate on 1000 yen trials. Normalized low-frequency power (nLF), normalized high-frequency power (nHF), and the ratio of low-frequency to high-frequency power (LF/HF) were calculated for each session on the fourth day and normalized to the first training session. In the scatter plots, each dot represents an individual result (after removing outliers, see “Methods” section). The change in nLF (**a**), an index of sympathetic dominance, but the not parasympathetic index nHF (**b**), was associated with poor performance. The black line in (**c**) represents the regression line and the shaded area is the 95% confidence interval.

The changes in HRV indices during each test session relative to the first training session on the fourth day were then calculated for 9 participants (5 were excluded due to electrode displacement or signal quality degradation due to body motion) and examined for correlations with 1000 yen trial performance. The results revealed a significant negative correlation between the change in nLF and performance in the 1000 yen condition (Fig. 4a; *r*(7) =  − 0.84, *p* = 0.004). However, no significant correlation was found between performance and nHF (Fig. 4b; *r*(7) =  − 0.16, *p* = 0.68) or LF/HF (Fig. 4c; *r*(7) =  − 0.57, *p* = 0.11). Moreover, there was no significant correlation between performance in any reward conditions other than the 1000-yen condition and the change in nLF (1 yen: *r*(7) =  − 0.12, *p* = 0.76; 10 yen: *r*(7) = 0.07, *p* = 0.86; 100 yen: *r*(7) = 0.33, *p* = 0.39).

### A second experiment replicated increased hand velocity with reward and the negative correlation between pre-movement BB-TB CCI and performance in highest reward trials

In the first experiment, reward increased the mean hand velocity in both HP and LP groups, but increased the variability of hand velocity only in the LP group. Moreover, reward increased the FCR-ECR CCI in both groups during movement but increased the pre-movement BB-TB CCI only in the LP group. There was also a negative correlation between task performance and the BB-TB CCI among LP participants on 1000 yen trials. Thus, poor performance on the highest reward trials among this subgroup may be explained by aberrant pre-movement co-contraction of the BB and TB.

**Figure 5:**
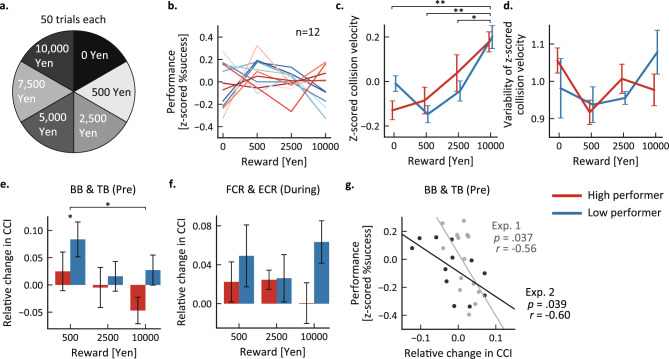
Experimental replication of the negative association between pre-movement BB-TR CCI and reward amount under higher reward conditions. Twelve participants practiced the velocity control task for three days and engaged in test sessions on the fourth day. (**a**) Six different larger rewards were offered with equal frequency during test sessions. (**b**) Task performance. The success rate was z-scored for each participant. Each line represents the performance of one individual, with blue lines representing participants whose performance worsened on 10,000 yen trials compared to 2500 yen trials (low performers) and red lines representing those who performed better on the highest reward trials (high performers). (**c**,**d**) Averaged hand velocity (**c**) and standard deviation of hand velocity (**d**) at the instant of ball collision. (**e**,**f**) Changes in CCI by monetary reward amount. Here, we analyzed only CCIs that differed between high and low performers in the main experiment, namely pre-movement (Pre) CCI of the BB-TB and movement (During) CCI of the FCR-ECR pair. Group results are presented as mean ± SEM. *p < 0.05; **p < 0.01. (**g**) Correlation between Pre-BB-TB CCI and task success rate in highest reward trials. The regression lines of the second experiment (black line) and the main experiment (gray line) are shown.

However, the first experiment had two limitations: the previously described inverted U-shaped relationship between reward amount and performance (“choking under pressure”) was not observed, and the degree of variance in statistical metrics among reward conditions differed due to the uneven number of trials (Fig. 2a). Therefore, we conducted a second experiment design to address these limitations while still providing evidence for reproducibility of the core findings (Fig. 5a). In this second experiment, the maximum reward was increased to apply more pressure, and the number of trials with each reward condition was equalized as in a previous study^18^. Note that although six reward conditions were selected in accord with Chib et al.^18^, only the three lowest and the highest reward conditions were incorporated in the statistical test because these trials should yield the greatest difference in psychological condition (pressure) and yield the most significant differences.

Twelve healthy adults were newly recruited for the second experiment, which mirrored the first in consisting of three training days followed by a fourth day of test sessions with monetary rewards (Table 3). As in the first experiment, task performance did not exhibit an inverted U-shaped relationship with reward amount (Fig. 5b). In addition, the increase in average hand velocity with reward amount was replicated (Fig. 5c; the main effect of reward: *F*(3, 30) = 10.1, *p* < 0.001). In contrast, the increased variability on the highest reward trials among the LP group was not replicated (Fig. 5d; interaction: *F*(3, 30) = 1.06, *p* = 0.38). The relationship between reward amount and muscle indices also differed from the first experiment in that pre-movement BB-TB CCI increased in both group on trials with the smallest reward of 500 yen (Fig. 5e; main effect of reward: *F*(3, 30) = 4.46, *p* = 0.011; interaction: *F*(3, 30) = 1.59, *p* = 0.21). Further, there was no main effect of reward on movement FCR-ECR CCI and no interaction effect between reward and performance group (Fig. 5f; the main effect of reward: *F*(3, 30) = 2.32, *p* = 0.10; interaction: *F*(3, 30) = 1.93, *p* = 0.15). However, as in the first experiment, there was a significant negative correlation between pre-movement BB-TB CCI and performance in the highest reward condition (10,000 yen) (Fig. 5g; *r*(10) =  − 0.60, *p* = 0.039), but not in the other reward conditions (500 yen: *r*(10) = 0.24, *p* = 0.46; 2500 yen: *r*(10) = 0.25, *p* = 0.43; Supplementary Fig. 3c,d).

**Table 3:** Performance, kinematics, and CCI in the second experiment.

Reward [yen]	Subgroup	Performance, [z-scored %success]	Z-scored collision velocity	Variability of z-scored collision velocity
Performance and kinematics				
1	HP	− 0.017 (0.19)	− 0.13 (0.10)	1.1 (0.082)
	LP		− 0.010 (0.086)	0.98 (0.20)
500	HP	0.074 (0.13)	− 0.086 (0.15)	0.92 (0.080)
	LP		− 0.15 (0.094)	0.94 (0.12)
2500	HP	1.7 × 10^−4^ (0.099)	0.038 (0.20)	1.0 (0.10)
	LP		− 0.043 (0.11)	0.96 (0.042)
10,000	HP	− 0.057 (0.18)	0.18 (0.11)	0.98 (0.10)
	LP		0.20 (0.13)	1.1 (0.14)

Reward [yen]	Subgroup	BB & TB (Pre)	FCR & ECR (During)	
CCI				
500	HP	0.025 (0.087)	0.022 (0.050)	
	LP	0.084 (0.078)	0.049 (0.078)	
2500	HP	− 4.8 × 10^−3^ (0.090)	0.025 (0.024)	
	LP	0.016 (0.067)	0.026 (0.059)	
10,000	HP	− 0.047 (0.059)	5.9 × 10^−4^ (0.051)	
	LP	0.027 (0.068)	0.063 (0.053)	

Values represent the mean and standard deviation.

## Discussion

This study examined the deterioration of motor task performance under high-reward conditions as well as the underlying muscle contraction patterns to identify biomarkers and targets for intervention. Greater reward value consistently increased movement velocity across all participants, but in addition increased the co-contraction of an antagonist muscle pair (BB and TB) during the pre-movement phase among the subset of participants showing a significant performance deficit on highest reward trials. Furthermore, across two separate experiments, muscle co-contraction before movement initiation were negatively correlated with performance level in only the highest reward condition. These findings indicate that training or other interventions for alleviating aberrant co-contraction of antagonistic muscle pairs prior to motor execution may improve performance under pressure.

The decline in motor performance on highest reward trials can be attributed to enhanced antagonist muscle activity, which impedes task execution. Co-contraction of antagonistic muscle pairs is essential for precise movements^25^ as it enhances stability^26^. However, increased activity of antagonist muscles reduces net torque and stiffens the joint, which may hinder the execution of smooth movements^27,28^. Excessive co-contraction might occur in high-reward conditions, which could further impede the intended movement. Notably, in our current motor task primarily centered on elbow control, the effect of co-contraction between the flexor BB and extensor TB muscle was especially pronounced.

Exploring the connection between the observed limb muscle co-contraction in this study and previously reported changes in brain activity induced by large rewards was a challenging and intriguing task. Notably, the current study and previous studies revealed a negative correlation between motor performance and the activity of limb muscles or the ventral midbrain (VM)^17^. Our findings illustrated that increased muscle co-contraction before movement initiation was negatively correlated with motor performance. A previous study found that high rewards were associated with increased activity of the VM, a region involved in motivation and behavioral arousal, and this increased activity was negatively correlated with motor performance^17^. In addition, a recent study involving monkeys demonstrated that VM activity can modulate the activities of descending motor pathways, including muscle responses^29^. Although a direct investigation into the relationships among rewards, VM activity, and muscle contraction has not been conducted, we infer that alterations in VM activity prompted by excessive rewards might influence motor commands, leading to aberrant muscle co-contraction and subsequent performance degradation.

The consistent increase in average movement velocity with rising reward indicates that the reward served as effective motivation. This observation is consistent with the findings of Summerside et al.^4^, who reported a similar positive relationship between reward value and reaching movement velocity among human participants and proposed that a larger reward warrants greater expenditure of effort. The observed changes in movement due to reward in our study are thus likely attributable to a similar increase in vigor.

Similar to the LP group, monkeys demonstrated a performance decline in a position control reaching task with high-value reward, but poor performance in this case was associated with an undershoot of reaching movements^21^. The apparent inconsistency between this undershoot on high-reward trials and the increased vigor observed with high reward in the present study may be explained by differences in the specific movement pattern required for task success. In the position control task employed by Smoulder et al.^21^, precise deceleration was required during the latter part of reaching movement, so undershoot occurred due to early or excessive deceleration. In contrast, the velocity control task used in our study allowed success without movement deceleration, thereby avoiding instances of reaching movement undershoot.

We also found that low task performance was associated with enhanced sympathetic activation as evidenced by HRV analysis. However, the negative correlation between the changes in nLF from training to test sessions and task success rate under the highest reward condition should be interpreted with caution. Previous studies have indeed reported that nLF is an indicator of sympathetic dominance^23,24^, suggesting that individuals with a higher sympathetic dominance during test sessions are more susceptible to performance decline on high-reward trials. Further, sympathetic nervous system activation is a core component of the body’s stress response, which may in turn interfere with motor performance. However, the relationships between HRV indices like nLF, nHF, and LF/HF, and autonomic nervous system activity have been questioned^30,31^. Therefore, follow-up studies should estimate sympathetic and parasympathetic tone using multiple physiological measures to confirm this association.

We conducted a second experiment to address some limitations of the first experiment and replicate the core findings. While an association between poor performance on high-value reward trials and pre-movement BB-TB was replicated, there were several differences. In the first experiment, there was a group × reward interaction effect on the variability in hand velocity and CCI of BB and TB muscles before movement initiation. However, this interaction effect was insignificant in the second experiment. If this discrepancy was not due to statistical chance (Type I error), it could be attributed to several factors. First, the greater frequency of highest reward trials in the second experiment may have influenced the results as a previous study of nonhuman primates reported that the rarity of reward and its value contribute to performance decrements^21^. Second, there was a difference in the reward ratio between the highest and second-highest reward conditions. In the first experiment, the highest reward was tenfold larger than the second-highest, whereas in the second experiment, the difference in the rewards was only fourfold. Third, in the second experiment, the test sessions included six reward conditions, each repeated 50 times, which may have reduced psychological pressure during the highest reward trials. Indeed, previous human studies have reported that repeated exposure to conditions inducing psychological pressure can mitigate the associated decline in motor performance^20^. Conducting another experiment considering inconsistencies in the experimental protocols between the two experiments might allow to establish connections between more kinematic or muscle activation patterns and the reduced performance.

The present study utilized CCI to quantify muscle co-contraction from surface EMG data due to its simplicity and validity. Despite the availability of alternative methods, such as estimating co-contraction by joint moments from EMGs and muscle–tendon lengths using anatomical models^32^ or assessing muscle activation overlap in EMG waveforms^33^, the complexity of the former approach precluded its use in our study. CCI has demonstrated a stronger correlation with joint stiffness on comparing CCI with the latter method^34^. However, as an important limitation, CCI only considers co-contraction between a single pair of muscles, and it might not fully address factors such as differences in muscle force capacities, potentially leading to less precise assessments of muscle co-contraction.

This study revealed reduced motor task performance on highest reward trials among a subset of participants and a significant negative correlation between pre-movement antagonistic muscle co-contraction and task performance on these highest reward trials. These findings suggest that reducing pre-movement co-contraction to an appropriate level may prevent performance declines in high-reward conditions. However, the significant correlation identified in this study was based on task success rates and averaged CCI for each specific reward condition, and so does not imply a direct causal relationship at the single-trial level. Moreover, given that co-contraction can enhance movement accuracy^25^, the impact of co-contraction on performance is potentially complex. Future investigations are required to demonstrate whether muscle co-contraction is a causal factor impeding motor performance or merely a surrogate marker allowing the prediction of performance decrements by excessive rewards that do not mitigate performance decrements by suppressing muscle co-contraction before movement initiation. This will support the viability of the proposed training, which intends to alleviate aberrant co-contraction of antagonistic muscle pairs before motor execution to improve performance under pressure.

## Methods

### Participants

A total of 26 individuals (mean ± SD, 22 ± 2.8 years of age, 9 females) participated in the experiments, with 14 participating in the main experiment and 12 in the second experiment. All participants were right-handed and reported no history of neurological or psychiatric diseases. The study was conducted in accordance with the Declaration of Helsinki and the research protocol was approved by the Ethics Committee of the Faculty of Science and Technology, Keio University (IRB approval number: 2021-115). All participants provided written informed consent.

### Experimental apparatus

A custom-designed C +  + program was used for both the motor velocity task and data acquisition. The task stimulus was presented on an LED monitor (1920 × 1080 pixels). Participants sat on a stool positioned 0.6 m away from the monitor while grasping the handle of a robotic manipulandum (Phantom Premium 1.5HF, SensAble Technologies, Wilmington, MA) with their right hand. The motor task involved manipulating a bar on the task screen via the robot manipulandum to strike a virtual ball toward a target. Participants were able to move the right hand in the up, down, left, and right directions only (no back and forth). The height of the stool was adjusted for each participant to align their eye level with the screen. Additionally, to ensure a stable body and arm posture throughout the experiment, participants rested their elbows on armrests, and the armrest height was adjusted to ensure the arm was horizontally aligned with the ground. This setup aligned with the bottom edge of the manipulandum, in which participants were required to continuously hold. Before movement initiation, participants were explicitly instructed to maintain straight wrists and avoid any flexion, extension, or pronation. However, wrist flexion and extension were allowed during the movement to facilitate natural task execution. Elbow and wrist postures were monitored during training sessions to verify adherence to these instructions and provided corrective guidance if needed. With these instructions and corrections during the training sessions, participants could maintain specified postures throughout testing sessions.

### Velocity control task

The velocity control task is illustrated in Fig. 1b. The task screen consisted of a yellow circle of radius 6 mm (representing the start position), a gray bar 25 mm in width and 100 mm in height (representing hand movement), a ball of radius 6 mm (initially white), and a green arch target. The target width was adjustable, allowing for modification of task difficulty. The ball was initially positioned at the center of the screen, with the target located 100 mm to the left and the starting position located 100 mm to the right.

At the beginning of each trial, participants moved the bar over the yellow circle and maintained this position. Following a random interval of 1–2 s, the ball changed color from white to red as a Go cue. Participants were required to move the bar with their right hand within 2 s of the Go cue and hit the ball within 0.8 s. Upon successful impact within the specified time limit, the motion of the ball was simulated based on hand velocity at the point of contact. The contact angle between the ball and bar influenced the direction of the ball’s movement. However, this had minimal effect on the success or failure of the task because of the goal’s wide width. Subsequently, the trial outcome (success or failure hitting the target) was displayed for 2 s along with the endpoint of the ball. A trial was deemed successful if the center of the ball fell within the target area (or a failure if the ball center stopped outside the target area). If the time limit was exceeded, a screen was displayed indicating that the attempt had failed. After performance feedback, participants returned their hand to the start position and the next trial began.

The endpoint of the ball *x* was determined by the function $x=k{v}^{2}$, where *v* represents the hand velocity upon hitting the ball, accounting for movements in the left–right and up–down directions, and *k* is the proportionality constant that was same across all participants. The ideal hand velocity required for the ball to reach the center of the target was *v* = 384 mm/s (*k* = 600 s^2^/mm) for training sessions on experimental days 1–3 and *v* = 444 mm/s (*k* = 800 s^2^/mm) on experimental day 4 (including one training session and four test sessions). Although the difference in velocity between days 1–3 and day 4 was unintended, statistical analysis indicated no detrimental effects on task success on day 4. Furthermore, EMG analysis was exclusively conducted using data collected on day 4, on which the target velocity remained consistent across all tests. Therefore, it is unlikely that the variation in target velocities across different days influenced the research objectives.

### Experimental protocol

The experiment consisted of two phases, training and testing, which were conducted over four consecutive days (Fig. 1c). Training took place from experimental day 1 through day 3 and the first session of experimental day 4. The test phase was conducted after the first session of day 4. Before the start of the experiment, participants received a briefing on the task. However, detailed instructions regarding the test were provided only at the beginning of day 4.

#### Training sessions

Each training session comprised 120 trials, and five sessions were performed each day from day 1 to day 3, and one session was performed on day 4. Task difficulty was adjusted individually for each subject to achieve an 80% success rate. This difficulty level was selected based on theoretical research indicating that learning efficiency is highest when training with approximately 80% success^35^. Target widths for training trials on days 1–3 were adjusted both within and between sessions starting from 35 mm at the beginning of session 1. Within-session adjustments occurred every 10 trials. Briefly, the success rate of the previous 10 trials was assessed, and if 100%, the target width was decreased by 1 mm, while if below 70%, the target width was increased by 1 mm. Otherwise, the target width remained constant. Between-session adjustments were conducted using data from the entire previous session. The differences between the endpoint of the ball and the center of the target were computed for all trials and sorted in ascending order. Then, target width for the next session was determined as the 80th percentile value of these differences.

The training session on day 4 served as preparation for the test sessions. Unlike previous training sessions, the target width was not adjusted within this final preparatory session. To determine the difficulty level, we calculated the endpoint errors from the center of the target for all trials on day 3 and obtained the average for each session. The sessions with the second and third lowest average errors were identified, and the endpoint errors in these sessions were arranged in ascending order. The target width for the training session on day 4 was then set as the 80th percentile value of the total endpoint errors. This approach aimed to mitigate the possibility of setting an excessively challenging target width for the test sessions due to unusually high performance on any single training session.

#### Test sessions

The task difficulty in test sessions was adjusted for each participant to achieve a 60% success rate. This difficulty level was based on empirical findings^18^ suggesting that performance decrements due to pressure often manifest in moderately difficult tasks with an approximate 60% success rate. The target width was fixed for all 4 test sessions and was determined following the same procedure as described for the training session on day 4 except that the measurements from this final training session were also combined with those from day 3.

During the test sessions, a monetary reward for success was set for each trial and presented for 2.5 s at the beginning (Fig. 2b). Upon successfully trial performance, the reward amount acquired was displayed on the screen along with endpoint feedback. The sum of rewards earned was not disclosed to participants until the conclusion of all test sessions. The accumulated rewards were recorded separately for each session, and one accumulated value was randomly selected as actual payment after the experiment. In addition, participants received a basic show-up fee of 6000 yen for the first experiment or 4000 yen for the second experiment.

The structure of test sessions differed between the first and second experiments. In the first (primary) experiment, each of the four test sessions consisted of 110 trials, while the second experiment involved five test sessions each comprising 60 trials. This change in session structure between the two experiments was introduced due to the difference in reward conditions. There were four reward conditions in the first experiment: 1, 10, 100, and 1000 yen. Out of the 110 trials in each session, 35 trials each were rewarded 1, 10, and 100 yen if successful, while only 5 trials were rewarded 1000 yen for success (Fig. 2a). In the second experiment, however, reward amounts of 0, 500, 2500, 5000, 7500, and 10,000 yen were equally assigned to trials (Fig. 5a). The reward for each trial was selected pseudo-randomly, except that the highest reward was never assigned to the first trial of a session.

### Data analysis

Data analysis was conducted using Python 3.9 (Python Software Foundation) and statistical analysis using JASP (version 0.17.1). Trials with reaction times from the Go cue to movement onset exceeding physiological limits (shorter than 100 ms) were excluded from the analysis^36,37^.

#### Analysis of task performance during test sessions

Hand velocity at the instant of ball strike and outcome (success or failure) were derived from hand position and velocity data collected at a sampling frequency of 1000 Hz. To account for variations in task skill among participants, performance metrics were z-scored. Success rate, average hand velocity, and standard deviation of hand velocity were computed for each reward condition. Participants were then categorized as HPs or LPs based on whether performance improved or declined, respectively, on 1000 yen trials compared to 100 yen trials. A two-way ANOVA was conducted with reward amount as the within-subject factor and performance group (HP or LP) as the between-subject factor. Prior to conducting ANOVA, we assessed the sphericity assumption using Mauchly’s test. If the sphericity assumption was violated, we applied the Greenhouse–Geisser correction to adjust for the degrees of freedom. If ANOVA yielded statistically significant main effects or a reward condition × group interaction, post-hoc t-tests were performed with Bonferroni correction to compare specific condition or group differences.

#### EMG signal collection and signal processing

Surface EMG signals were measured on day 4 from six upper limb muscles, the BB, TB, FCR, ECR, FDS, and EDC, using wireless EMG devices (mini Wave Infinity and Pico, COMETA Systems, Milan, Italy). Signals were digitized at a sampling frequency of 1000 Hz using a CONTEC Analog IO board (AIO-163202F-PE) and stored on a computer for off-line analyses. According to the EMG device manufacturer’s documentation, a consistent delay of 14 ms occurs between the measurement of EMG signals and their analog output. To compensate for this delay, the timing of EMG data was adjusted during the off-line analysis, ensuring accurate temporal alignment with kinematic data. Subsequently, the digitized EMG signals were high-pass filtered with a Butterworth filter (4th order, 40 Hz cutoff frequency) to remove movement artifacts associated with the execution of the velocity control task^34^. The filtered EMG signals in the first trial of each session were excluded from further analysis because it included muscle activity derived from non-task-related body movement.

Artifact-free EMG signals were full-wave rectified and smoothed using a low-pass Butterworth filter (4th order, 20 Hz cutoff frequency). These processed EMG records were then divided into three pairs, BB and TB, FCR and ECR, and FDS and EDC, and the CCI was computed for each pair using the following equation:

$$CCI(t) = \frac{Lo(t)}{Hi(t)}(Lo(t)+Hi(t))$$

In this equation, *Lo*(*t*) represents the smaller value of each pair, and *Hi*(*t*) corresponds to the other value at a given time *t*^38^. The average CCI was calculated for each muscle pair at two time intervals, the pre-movement period spanning 1000 ms before the Go cue, and the movement period from movement onset to the ball hit. For each CCI, a two-way ANOVA followed by post-hoc t-tests was conducted using the same procedure for task performance analysis. Subsequently, a correlation analysis between performance and CCI was performed. Prior to the correlation analysis, outlier correction was applied by winsorizing CCI and performance data across participants, using a criterion of two standard deviations from the mean.

#### ECG recording and processing

In the first experiment on day 4, the ECG was measured simultaneously with task performance using a portable bioelectric amplifier (EBA-100, UNIQUE MEDICAL, Tokyo, Japan) and three electrodes placed on the right clavicle, left clavicle (ground), and left abdomen, allowing for a typical Lead II configuration while minimizing motion artifacts. The hardware filter settings were low cutoff frequency of 0.15 Hz and a high cutoff frequency of 240 Hz.

Since HRV analysis requires a minimum 1–2 min of ECG recording^23^, the analysis was performed on a session-by-session basis. The NeuroKit2 Python library^39^ was used for R-peak detection, R-R interval calculation, and HRV analysis in the frequency domain. The relative changes in HRV indices for each test session were normalized to the corresponding indices measured during the first training session on day 4 and averaged across all test sessions. Excluding those indices that increased by more than fivefold from training to test sessions, the remaining indices were then winsorized across participants to correct for outliers, employing a winsorizing criterion of two standard deviations from the mean, and Pearson correlation coefficients were calculated between each index and z-scored performance in the 1000 yen condition. *p* = 0.05 (corrected) was considered a statistically significant correlation.

### Supplementary Information

Supplementary Information.

## Data Availability

Data to generate all plots in Figs. 1, 2, 3, 4, and 5 can be found at https://osf.io/xgpjm/. The other data (e.g., raw data) will be provided upon request to the corresponding authors.
