# Supplementary material for: Pre-movement muscle co-contraction associated with motor performance deterioration under high reward conditions
Source: Sci Rep. 2024 Jul 19;14:16710. doi: 10.1038/s41598-024-67630-5 (PMC11271558; doi:10.1038/s41598-024-67630-5)
Supplement: Supplementary file 1 — Supplementary Information. [file 41598_2024_67630_MOESM1_ESM.pdf]

Pre-movement muscle co-contraction associated with motor performance deterioration under high reward conditions

Authors

Naoki Senta[1], Junichi Ushiba[2], Mitsuaki Takemi[1*]

Affiliations

[1] Graduate School of Science and Technology, Keio University, Yokohama, Japan

[2] Faculty of Science and Technology, Keio University, Yokohama, Japan

***Corresponding author: Mitsuaki Takemi**

Address: 3-14-1 Hiyoshi, Kohoku-ku, Yokohama, Kanagawa, 223-8522, Japan

Tel/Fax: +81-45-566-1678; E-mail: mitsuaki1988@me.com

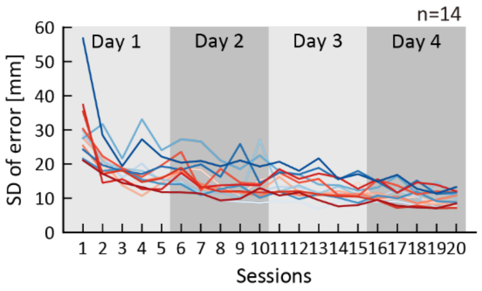

Supplementary figure 1. Time course of standard deviation (SD) of endpoint error calculated over trials within a session.

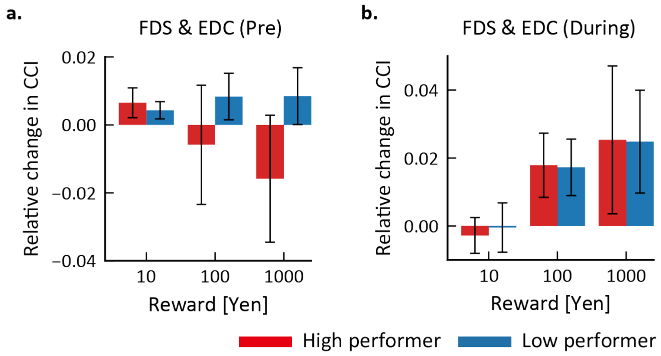

Supplementary figure 2. CCI modulation with the amount of monetary reward. We compared CCIs of flexor digitorum superficialis (FDS) and extensor digitorum communis (EDC) between high and low performers in two analysis periods (Pre mov. and During mov.).

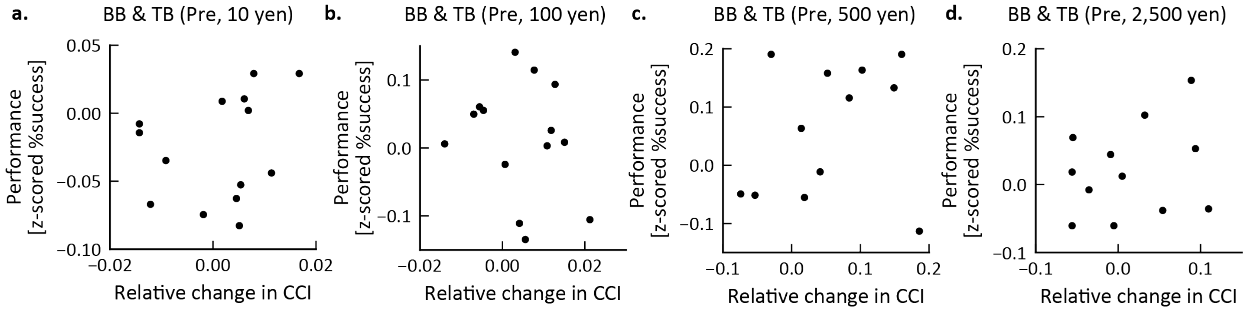

Supplementary figure 3. Scatter plots of CCI versus task success rate for (A) 10 yen, (B) 100 yen trials in the first experiment and (C) 500 yen, (D) 2,500 yen trials in the second experiment. Neither correlation was not statistically significant. Levels of significance: $\alpha = 0.05$.

Supplementary table 1. CCI modulation with the amount of monetary reward and participants' subgroup.

Pre movement.

Cases	FDS-EDC	FCR-ECR	BB-TB
Reward	$F(1.94, 23.22) = 0.33$	$F(1.31, 15.67) = 0.91$	$F(1.54, 18.44) = 2.63$
Subgroup	$F(1, 12) = 1.12$	$F(1, 12) = 0.02$	$F(1, 12) = 3.35$
Reward × Subgroup	$F(1.94, 23.22) = 0.92$	$F(1.31, 15.67) = 1.84$	$F(1.54, 18.44) = 4.61^{*}$

*$p < .05$

During movement.

Cases	FDS-EDC	FCR-ECR	BB-TB
Reward	$F(1.52, 18.18) = 3.67$	$F(1.65, 19.74) = 6.16^{*}$	$F(1.58, 18.96) = 2.08$
Subgroup	$F(1, 12) = 9.90\times10^{-4}$	$F(1, 12) = 1.48$	$F(1, 12) = 1.57$
Reward × Subgroup	$F(1.52, 18.18) = 0.01$	$F(1.65, 19.74) = 2.54$	$F(1.58, 18.96) = 0.47$

*$p < .05$

Post-Hoc test of BB-TB CCI in pre-movement.

Subgroup, Reward (Yen)	Subgroup, Reward (Yen)	t
HP, 1	LP, 1	$t = 1.36\times10^{-15}$
	HP, 10	$t = 0.21$
	HP, 100	$t = -0.85$
	HP, 1,000	$t = 0.36$
LP, 1	LP, 10	$t = -0.33$
	LP, 100	$t = -0.35$
	LP, 1,000	$t = -3.86^{*}$
HP, 10	LP, 10	$t = -0.52$
	HP, 100	$t = -1.06$
	HP, 1,000	$t = 0.15$
LP, 10	LP, 100	$t = -0.02$
	LP, 1,000	$t = -3.53^{*}$
HP, 100	LP, 100	$t = 0.48$
	HP, 1,000	$t = 1.21$
LP, 100	LP, 1,000	$t = -3.51^{*}$
HP, 1000	HP, 1,000	$t = -4.05^{**}$

*$p < .05$, **$p < .01$, corrected

Post-Hoc test of FCR-ECR CCI during movement.

Reward (Yen)	Reward (Yen)	t
1	10	$t = -0.21$
	100	$t = -1.62$
	1,000	$t = -3.81^{*}$
10	100	$t = -1.41$
	1,000	$t = -3.60^{*}$
100	1,000	$t = -2.19$

*$p < .05$, corrected
